# Longitudinal Analysis of One-Carbon Metabolism-Related Metabolites in Maternal and Cord Blood of Japanese Pregnant Women

**DOI:** 10.3390/nu16111765

**Published:** 2024-06-04

**Authors:** Yoshinori Kubo, Hideoki Fukuoka, Kumiko Shoji, Chisato Mori, Kenichi Sakurai, Masazumi Nishikawa, Kyoichi Oshida, Yuichiro Yamashiro, Terue Kawabata

**Affiliations:** 1 Faculty of Nutrition, Kagawa Nutrition University, 3-9-21 Chiyoda, Sakado 350-0288, Saitama, Japan; shoji.kumiko@eiyo.ac.jp (K.S.); kawabata@eiyo.ac.jp (T.K.); 2 Division of Anatomy and Cell Biology, Department of Anatomy, Shiga University of Medical Science, Seta Tsukinowa-cho, Otsu 520-2192, Shiga, Japan; 3 Department of Perinatal Mesenchymal Stem Cell Research, Fukushima Medical University School of Medicine, 1 Hikarigaoka, Fukushima 960-1295, Fukushima, Japan; fukuokah@fmu.ac.jp; 4 Department of Bioenvironmental Medicine, Graduate School of Medicine, Chiba University, 1-8-1 Inohana, Chuo-ku, Chiba 260-8670, Chiba, Japan; cmori@faculty.chiba-u.jp; 5 Department of Sustainable Health Science, Center for Preventive Medical Sciences, Chiba University, 1-33 Yayoi-cho, Inage-ku, Chiba 263-8522, Chiba, Japan; 6 Department of Nutrition and Metabolic Medicine, Center for Preventive Medical Sciences, Chiba University, 1-33 Yayoi-cho, Inage-ku, Chiba 263-8522, Chiba, Japan; sakuraik@faculty.chiba-u.jp; 7 Department of Food Management, School of Food, Agricultural and Environmental Sciences, Miyagi University, 2-2-1 Hatadate, Taihaku-ku, Sendai 982-0215, Miyagi, Japan; nishikaw@myu.ac.jp; 8 Faculty of Beauty & Wellness, Professional University of Beauty & Wellness, 3-9-3 Ushikubo, Tsuzuki-ku, Yokohama 224-0012, Kanagawa, Japan; kyoichi.oshida@gmail.com; 9 Probiotics Research Laboratory, Graduate School of Medicine, Juntendo University, 2-9-8-3F, Hongo, Bunkyo-ku, Tokyo 113-0033, Japan; yamasiro@juntendo.ac.jp

**Keywords:** one-carbon metabolism, 5-methyltetrahydrofolate, betaine, methionine, homocysteine, *S*-adenosylmethionine, *S*-adenosylhomocysteine, transsulfuration pathway, cysteine, pregnant woman

## Abstract

One-carbon metabolism (OCM) is a complex and interconnected network that undergoes drastic changes during pregnancy. In this study, we investigated the longitudinal distribution of OCM-related metabolites in maternal and cord blood and explored their relationships. Additionally, we conducted cross-sectional analyses to examine the interrelationships among these metabolites. This study included 146 healthy pregnant women who participated in the Chiba Study of Mother and Child Health. Maternal blood samples were collected during early pregnancy, late pregnancy, and delivery, along with cord blood samples. We analyzed 18 OCM-related metabolites in serum using stable isotope dilution liquid chromatography/tandem mass spectrometry. We found that serum S-adenosylmethionine (SAM) concentrations in maternal blood remained stable throughout pregnancy. Conversely, S-adenosylhomocysteine (SAH) concentrations increased, and the total homocysteine/total cysteine ratio significantly increased with advancing gestational age. The betaine/dimethylglycine ratio was negatively correlated with total homocysteine in maternal blood for all sampling periods, and this correlation strengthened with advances in gestational age. Most OCM-related metabolites measured in this study showed significant positive correlations between maternal blood at delivery and cord blood. These findings suggest that maternal OCM status may impact fetal development and indicate the need for comprehensive and longitudinal evaluations of OCM during pregnancy.

## 1. Introduction

**Figure 1:**
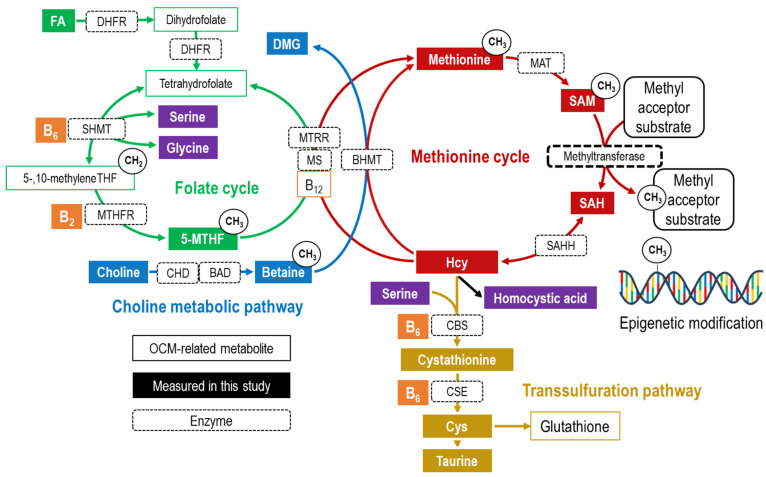
Overview of one-carbon metabolism. OCM-related metabolites are indicated by rectangular boxes, with the folate cycle in green, the choline metabolic pathway in blue, the methionine cycle in red, and the transsulfuration pathway in yellow. Other vitamins are shown in orange, and amino acids and others are shown in purple. The filled rectangular boxes indicate OCM-related metabolites measured in this study. Each arrow represents a biochemical reaction, and the dotted rectangle on the arrow is labeled with the first letter of the enzyme catalyzing the reaction. Abbreviations: 5-MTHF, 5-methyltetrahydrofolate; B_12_, cobalamin/methylcobalamin; B_2_, riboflavin; B_6_, pyridoxal phosphate (pyridoxine/pyridoxal/pyridoxamine); BAD, betaine aldehyde dehydrogenase; BHMT, betaine–homocysteine methyltransferase; CBS, cystationine-β synthase; CHD, choline dehydrogenase; CSE, cystathionine γ-lyase; Cys, cysteine; DMG, dimethylglycine; FA, folic acid; Hcy, homocysteine; MAT, methionine adenosyltransferase; MS, methionine synthase; MTHFR, methylenetetrahydrofolate reductase; MTRR, methionine synthase reductase; SAH, S-adenosylhomocysteine; SAHH, *S*-adenosylhomocysteine hydrolase; SAM, *S*-adenosylmethionine; SHMT, serine hydroxymethyltransferase; THF, tetrahydrofolate. This metabolism diagram is reprinted from our previous paper [8].

Adverse environments during the periconceptional, fetal, and early postnatal periods increase the risk of developing noncommunicable diseases, leading to the concept of Developmental Origins of Health and Disease (DOHaD) [1,2]. According to the DOHaD theory, abnormal epigenetic modifications during developmental stages are considered new risk factors for noncommunicable diseases [1,2,3]. One-carbon metabolism (OCM) plays a crucial role in epigenetic modifications that regulate gene expression. The methyl group transfer from S-adenosylmethionine (SAM), which is synthesized through OCM, to histone or DNA causes epigenetic modifications and changes to S-adenosylhomocysteine (SAH) after transmethylation (Figure 1) [4]. Understanding OCM during pregnancy, when drastic epigenetic remodeling occurs, is vital for DOHaD research. OCM is a complex and interconnected network encompassing the folate cycle, choline metabolic pathway, methionine cycle, and transsulfuration pathway (Figure 1) [4]. Additionally, maternal OCM dynamics change during pregnancy [5,6,7]. Therefore, a comprehensive and longitudinal understanding of OCM during pregnancy is required.

The exact role of SAM and SAH as blood biomarkers during pregnancy remains unclear [9]. Although several studies have measured SAM and SAH levels in maternal and cord blood during pregnancy, most studies have been limited to a single time point [10,11,12,13,14,15,16,17]. A study in the U.S. examined longitudinal changes in SAM and SAH during pregnancy in healthy pregnant women; however, this study might not reflect typical physiological conditions because the participants were provided with supplements containing nutrients affecting OCM [18].

In our previous study, we measured serum OCM-related metabolite concentrations in maternal and cord blood and reported the longitudinal distribution of 5-MTHF, folic acid (FA), indicating that FA was circulating in an unmetabolized state, and total homocysteine (tHcy), along with their associations between maternal and cord blood [19]. In this study, we have investigated the choline metabolic pathway, the methionine cycle (including SAM and SAH), and the transsulfuration pathway. We also examined the changes in the longitudinal distribution of OCM-related metabolites in maternal blood serum, explored the relationship between maternal blood at birth and cord blood in these metabolites, and analyzed cross-sectional relationships between OCM-related metabolites in maternal blood at different blood collection periods and cord blood.

In our previous study, we observed that 5-MTHF levels decreased with advancing gestational age [19]. Furthermore, we hypothesized that 5-MTHF status may be linked to metabolic fluxes in the choline metabolic pathway, SAM, and transsulfuration pathway based on studies in nonpregnant young women [8]. In this study, we investigated whether similar associations exist during pregnancy.

## 2. Materials and Methods

### 2.1. Study Design

This longitudinal study involved pregnant women and their children, using the detailed methods reported in our previous publication [19]. Maternal blood serum samples were collected during early pregnancy (mean 11.4 weeks, standard deviation ± 0.8), late pregnancy (mean 28.4 ± 1.0 weeks), and delivery (mean 39.5 ± 1.1 weeks). Cord blood was drawn from the umbilical cord artery at the time of birth. We analyzed OCM-related metabolites at each of these blood collection time points.

This study was conducted in accordance with the Declaration of Helsinki. The study protocol was approved by the Biomedical Research Ethics Committee of the Graduate School of Medicine, Chiba University (ID: 451, 8 November 2013; ID: 462, 4 December 2013; ID: 502, 28 May 2014), the Ethics Review Committee for Human Genome/Gene Analysis Research, Waseda University (ID: 2013-G002 (3), 13 November 2015), and the ethics review committee of Kagawa Nutrition University (ID: 67, 6 July 2016).

### 2.2. Participants

This study represents a subset of participants from the Chiba Study of Mother and Child Health (C-MACH) [20], a birth cohort investigation. Recruitment of pregnant women from C-MACH was conducted from February 2014 to June 2015 who were less than 13 weeks of gestation and attended two hospitals in Chiba Prefecture and one hospital in Saitama Prefecture (Aiwa Hospital). The inclusion criteria were determined by random selection among the consenting candidates. In cases of miscarriage, stillbirth, withdrawal, or transfer to another hospital, follow-up was terminated.

The participants included in this study overlapped with those from our initial report and were drawn from the C-MACH cohort, which initially comprised 434 pregnant women. Of these, 146 mothers and their children visited Aiwa Hospital in Saitama, Japan, where FA fortification of cereals is not mandatory [19]. We have already confirmed that participants in this study had a lower 5-MTHF status than pregnant women in countries where FA fortification of cereals is mandatory [19].

### 2.3. Information on Mothers and Children

Maternal lifestyle and anthropometric data were obtained from self-administered questionnaires distributed during early and late pregnancy [19]. Medical data for mothers and children were collected from hospital medical records [19].

### 2.4. Measurement of Serum OCM-Related Metabolites

We previously reported a detailed method for measuring OCM-related metabolites in serum [8,19]. In brief, we quantified 18 OCM-related metabolites [5-MTHF, folic acid, choline, betaine, dimethylglycine (DMG), methionine, SAM, SAH, total tHcy, cystathionine, total cysteine (tCys), taurine, serine, glycine, riboflavin, pyridoxamine, and pyridoxine] in serum using stable isotope dilution liquid chromatography/tandem mass spectrometry. If a peak could not be detected in a sample or if the signal-to-noise ratio was >10, the concentration was set to 0, defined as below the limit of quantitation.

### 2.5. Statistical Analysis

Continuous variables are presented as a median and interquartile range because of the skewed distribution of serum OCM-related metabolites. Consistent with our previous report [8], we used specific ratios as indicator of activity of the metabolic pathway (substrate to product concentration ratios): the betaine/DMG ratio for indicator of betaine–homocysteine methyltransferase (BHMT) pathway [21], the SAM/SAH ratio for indicator of methyltransferase pathway [22], and the tCys/tHcy ratio for indicator of the metabolic pathway in the transsulfuration pathway flux (cystathionine β-synthase and cystathionine γ-lyase) [23]. We also measured the cord blood/maternal blood metabolite ratio at birth, indicating the number of times the concentration of metabolites in cord blood corresponds to that in maternal blood at birth.

A Wilcoxon signed-rank test was used to compare OCM-related metabolites in maternal serum at three time periods: early pregnancy, late pregnancy, and delivery (*n* = 113). Bonferroni correction was used to adjust for multiple comparisons (*p* < 0.05/3 = 0.0167). Differences between maternal blood at delivery and cord blood were assessed using the Wilcoxon signed-rank test (*n* = 114). Spearman’s rank correlation coefficient was used to evaluate the correlation between these two variables. The significance level was set at *p* < 0.05 (a two-tailed test). All statistical analyses were performed using JMP^®^ Pro version 17.0.0 (SAS Institute, Tokyo, Japan).

## 3. Results

### 3.1. Characteristics of the Study Population

The final analysis included 146 pregnant women who initially consented to participate in the study during early pregnancy. However, some participants dropped out during the study period, resulting in blood samples being analyzed from 131 women in late pregnancy, 116 women at delivery, and 121 umbilical cord blood samples [19]. The characteristics of pregnant women and their children are identical to those described in our previous report [19]. In brief, the participants were slightly older (mean age 32.3 ± 4.6 years) and had higher household incomes than the representative Japanese birth cohort population [24]. Most pregnant women in this study were non-smokers and nondrinkers. The population of healthy pregnant women and their children had lower rates of preterm births (1.7%) and low birth weight (3.3%) than the representative Japanese population [24].

### 3.2. Serum Concentrations of OCM-Related Metabolites across Blood Sampling Periods

Table 1 presents the distribution of serum OCM-related metabolite concentrations in maternal and cord blood, along with the changes observed across different blood sampling periods. Betaine and riboflavin concentrations significantly decreased during late pregnancy and at birth compared with early pregnancy. Conversely, choline, SAH, and serine concentrations significantly increased with advancing gestational age in the order of early, late, and at birth. Similarly, DMG and glycine concentrations significantly increased at birth compared with early and late pregnancy, and cystathionine concentrations significantly increased in late pregnancy and at birth compared with early pregnancy. Moreover, tCys and taurine concentrations showed a significant decrease in late pregnancy compared to early pregnancy and at birth. Methionine, SAM, pyridoxamine, and pyridoxine concentrations remained stable during pregnancy. Homocysteic acid was undetectable in all samples. The concentrations of 5-MTHF, FA, and tHcy were previously reported [19]. The betaine/DMG and SAM/SAH ratios, which are indicative of OCM enzyme activity, decreased significantly with advancing gestational age. In contrast, the tHcy/tCys ratio increased significantly with advancing gestational age.

### 3.3. Relationship between Maternal Blood at Birth and Cord Blood in OCM-Related Metabolites

#### 3.3.1. Comparison of Serum OCM-Related Metabolite Concentrations between Maternal Blood at Birth and Cord Blood

Table 1 shows the comparison of serum OCM-related metabolite concentrations between maternal blood at birth and cord blood. Serum concentrations of 5-MTHF, choline, betaine, DMG, methionine, SAM, SAH, cystathionine, taurine, serine, glycine, riboflavin, pyridoxamine, and pyridoxine were significantly higher in cord blood than in maternal blood at birth. Conversely, concentrations of tHcy and tCys were significantly lower in cord blood than in maternal blood at birth.

#### 3.3.2. Correlation of Serum OCM-Related Metabolite Concentrations between Maternal and Cord Blood at Birth

Table 2 shows the correlation results for OCM-related metabolite concentrations between maternal and cord blood at birth. Most OCM-related metabolites in serum showed significant positive correlations between maternal and cord blood at birth. However, taurine also exhibited a trend toward a positive correlation (ρ = 0.169, *p* = 0.0730).

### 3.4. Cross-Sectional Relationships between OCM-Related Metabolites in Maternal Blood at Each Blood Collection Period and Cord Blood

Table 3 shows the correlation matrix of serum 5-MTHF, betaine concentration, and betaine/DMG ratio with serum SAM, SAH, tHcy concentration, and tHcy/tCys ratio for maternal blood at each blood sampling period and cord blood, respectively. Other correlation matrices are shown in Supplementary Table S2.

#### 3.4.1. Correlation between Serum 5-MTHF or Betaine Concentration and SAM Concentration

Serum 5-MTHF or betaine concentrations showed significant positive correlations with SAM concentrations in maternal blood at all blood collection periods and in cord blood. Conversely, no consistent correlation was observed for SAH (Table 3).

#### 3.4.2. Longitudinal Changes in Correlation Coefficients for Homocysteine Metabolism during Pregnancy

The negative correlation coefficients between the betaine/DMG ratio and tHcy in maternal blood serum became progressively stronger with advancing gestational age (Table 3).

#### 3.4.3. 5-MTHF or Betaine Concentration Associated with the tHcy/tCys Ratio

During all blood sampling periods, negative correlation coefficients were observed between serum 5-MTHF concentration and the tHcy/tCys ratio, which were stronger than the correlations between 5-MTHF concentration and tHcy concentration alone (Table 3). Similarly, the negative correlation coefficient between serum betaine concentration and the tHcy/tCys ratio was stronger than that of tHcy concentration alone during all blood sampling periods (Table 3).

## 4. Discussion

In this study, we analyzed OCM-related metabolites in maternal and cord blood samples from healthy Japanese pregnant women, where FA fortification with cereal grains was not practiced. Our study is the first to longitudinally measure metabolites of the folate cycle, including 5-MTHF, the choline metabolic pathway, the methionine cycle (including SAM and SAH), and the transsulfuration pathway in maternal and cord blood.

In this study, serum SAM concentrations in maternal blood did not change significantly during pregnancy. However, SAH concentrations increased with advancing gestational age, and there were positive correlations between maternal blood at birth and cord blood. The tHcy/tCys ratio increased significantly with advancing gestational age. Furthermore, the tHcy/tCys ratio demonstrated a stronger negative correlation with 5-MTHF or betaine concentration compared to its correlation with tHcy concentration alone.

In this study, maternal blood serum concentrations of choline and DMG increased with advancing gestational age, whereas betaine concentrations decreased (Table 1). These findings are consistent with previous studies that reported similar trends of increases in choline [25,26,27,28,29] and DMG [25,28,30] and decreases in betaine concentrations during pregnancy [25,27,28,30]. Therefore, the participants exhibited choline metabolism patterns that aligned with those of previous studies. The increase in free choline in maternal blood during pregnancy is influenced by the increased expression and activity of phosphatidylethanolamine *N*-methyltransferase, which increases estrogen levels, leading to enhanced phosphatidylcholine production [31,32]. The decline in betaine levels during pregnancy may be attributed to increased expression of BHMT, which is influenced by estrogen [33], along with increased use of betaine for Hcy remethylation [25].

Furthermore, in our study, the betaine/DMG ratios were negatively correlated with tHcy levels in maternal and cord blood at all sampling periods, and these correlations strengthened with advancing gestational age (Table 3). Previously, we reported that in nonpregnant young women, when serum 5-MTHF concentrations in young women were divided into high-and low-MTHF groups by median, the low 5-MTHF group showed a possible enhancement of betaine-mediated Hcy metabolism compared with the high 5-MTHF group [8]. As gestation progresses, maternal serum 5-MTHF levels decrease [8]. In addition to 5-MTHF, previous studies have shown that cobalamin, a cofactor for the enzyme methionine synthase, which remethylates homocysteine by methyl group donation of 5-MTHF, decreases maternal blood levels and increases homocysteine as the pregnancy progresses [34]. When 5-MTHF and cobalamin are deficient, betaine is required to compensate for the lack of methyl groups, which is necessary for Hcy remethylation [28]. Previous studies suggest that a low-folate diet decreases the expression of methionine synthase in mice’s liver [35], and reduced SAM levels activate BHMT in rat liver [36]. Therefore, decreasing maternal 5-MTHF status with advancing gestational age may increase Hcy remethylation by BHMT, compensating for lower serum 5-MTHF concentrations.

In this study, serum SAM concentrations were significantly positively correlated with 5-MTHF and betaine concentrations in maternal blood across all collection periods (Table 3). These findings are consistent with those of our previous report on serum OCM-related metabolites in nonpregnant young women [8]. The decrease in serum 5-MTHF and betaine concentrations as pregnancy progresses is presumed to affect SAM concentrations because of decreased Hcy remethylation (Table 1). However, in this study, SAM concentrations were maintained constant during pregnancy (Table 1). SAM levels may be tightly regulated through various mechanisms, including allosteric inhibition of methylenetetrahydrofolate reductase [37,38,39], allosteric activation of cystathionine β-synthase [38,40], feedback regulation of methionine adenosyltransferase [41,42], and BHMT inhibition [43]. Notably, serum SAM concentrations showed significant positive correlations across all blood sampling periods (early pregnancy, late pregnancy, and at birth), suggesting that individuals with low serum SAM concentrations during pregnancy may consistently exhibit lower concentrations (Supplementary Figure S1).

Our study showed that maternal serum SAH and tHcy levels increased with advancing gestational age (Table 1), suggesting reversible metabolism between SAH and tHcy [44,45], where higher SAH levels may be associated with elevated serum tHcy levels. Moreover, the SAM/SAH ratio decreased with advancing gestational age. This decline could be attributed to the relative increase in SAH compared with the maintenance of SAM homeostasis [46,47]. However, in a previous study that measured plasma OCM-related metabolites in maternal blood at less than 12 weeks’ gestation and at birth in pregnant women in the United States who were continuously using supplements containing FA and other OCM-related nutrients [18], plasma concentrations of 5-MTHF and Hcy did not change significantly with advancing gestational age, and SAM also remained unchanged. Nevertheless, a significant increase in SAH was observed. Therefore, SAH levels may increase with advancing gestational age, regardless of the intake of supplements containing OCM-related nutrients. A previous study of methionine dynamics in healthy pregnant women in the United States using isotope tracers showed higher metabolic flux through the transsulfuration pathway in early pregnancy than in late pregnancy, with transmethylation being higher in late pregnancy than in early pregnancy [5]. Therefore, our findings indicate that the methyltransferase reaction, which increases with advancing gestational age, may contribute to increased serum SAH concentrations. Notably, SAH concentrations in maternal blood at birth did not correlate with those in early or late pregnancy, suggesting that factors other than OCM could have influenced the increase in SAH concentrations at birth (Supplementary Figure S1). Intracellular accumulation of SAH strongly inhibits the SAM-dependent methyltransferase reaction [48,49], highlighting the need for future studies to determine the potential adverse effects of increased SAH during pregnancy on fetal development.

In this study, serum methionine concentrations, the precursor of SAM, did not differ significantly between early and late pregnancy and maternal blood at birth (Table 1). Similar results have been reported in other prospective cohort studies [29,50,51,52]. Methionine is derived from protein catabolism in addition to OCM [53], suggesting that methionine may be preferentially homeostatic compared with other amino acids (serine, glycine, and cysteine) [54].

The maternal transsulfuration pathway is crucial for supplying cysteine, taurine, sulfate, and hydrogen sulfide to the fetus, along with decreasing Hcy [53,55,56]. In this study, serum tHcy concentrations in maternal blood increased significantly with advancing gestational age (Table 1). However, these results appear contradictory because theoretically, Hcy should decrease due to changes in OCM enzyme activity caused by increased estrogen during pregnancy, along with increased maternal circulating blood volume and decreased albumin levels [6]. Moreover, previous studies have reported a decrease in tHcy from early to mid-pregnancy [27,28,57,58,59]. Conversely, some studies have indicated an increase in tHcy from mid to late pregnancy [28,29,34,58,60,61,62,63]. Therefore, this study did not include measurements during mid-pregnancy, which may have missed the potential decrease in tHcy during this period.

Serum tCys decreased significantly in late pregnancy in this study (Table 1), but results from previous studies are inconsistent. Some studies have reported a decrease from early to mid-pregnancy followed by a plateau [51], a decrease from early to mid-pregnancy with a slight increase from mid to late pregnancy [58], a decrease from mid to late pregnancy [64], and in some cases, no change [29]. Further studies are required to systematically investigate changes in tCys levels during pregnancy.

The tHcy/tCys ratio increased significantly with advancing gestational age (Table 1). This ratio serves as an indicator of enzyme activity of the metabolic pathway in the transsulfuration pathway, suggesting a potential decrease in transsulfuration pathway flux as pregnancy progresses. This finding is consistent with that of a previous study [5]. In this study, the negative correlation coefficient between serum 5-MTHF or betaine concentration and the tHcy/tCys ratio was stronger than that of tHcy concentration alone. Similar results were previously reported in nonpregnant young women [8]. Therefore, higher concentrations of 5-MTHF or betaine may indicate lower Hcy and higher Cys concentrations. According to a mathematical model from an in silico study, increased flux in the transsulfuration pathway is because of allosteric activation of CBS caused by elevated levels of 5-MTHF, SAM, or betaine [65]. In summary, 5-MTHF and betaine levels may influence transsulfuration flux, and the tHcy/tCys ratio is a sensitive indicator of 5-MTHF and betaine levels compared with tHcy alone. Further studies are warranted to explore the clinical significance of activating the sulfur transfer pathway with 5-MTHF and betaine.

Moreover, most OCM-related metabolites measured in this study showed significant positive correlations between maternal blood at birth and cord blood, indicating that fetal OCM status reflects maternal OCM status (Table 2). Additionally, the concentrations of OCM-related metabolites in cord blood were higher than those in maternal blood at birth, except for FA, tHcy, and tCys (Table 1). 5-MTHF [66], choline [66], methionine [67], cysteine [67], glycine [67], serine [67], Hcy [68,69], taurine [55], and vitamin B_2_ [70] are actively transported from the mother to the fetus via the placenta. However, few associations between maternal and fetal SAM or SAH status have been reported. A previous study involving 24 pregnant women in the U.S. demonstrated a significant positive correlation in plasma SAH concentrations between maternal blood and cord blood at birth (similar to plasma SAM concentrations between maternal blood and cord blood in early pregnancy), partially supporting our findings [18].

However, whether the positive correlation between the concentrations of OCM-related metabolites in maternal blood at birth and cord blood is primarily due to placental transport from the mother to the fetus or if similar metabolism occurs within the placenta or on the fetal side as in the mother remains uncertain.

In summary, OCM is complex and requires comprehensive longitudinal evaluation due to drastic changes during pregnancy. Additionally, maternal OCM status may influence fetal OCM. Future studies are required that can assess maternal OCM status comprehensively and longitudinally during pregnancy and investigate its association with fetal epigenetic modifications.

The limitations of this study have been reported in our previous report [19]. Briefly, the study population was recruited from a single hospital, potentially introducing sampling bias. Additionally, the participants were not in a fasting state at the time of blood collection, which might have influenced the results. Genetic polymorphisms related to OCM and vitamin B_12_ were not considered in this analysis. Furthermore, serum OCM-related metabolites may not always directly reflect the OCM status in organ cells [71,72]. Metabolite concentration ratios do not directly reflect enzyme activity or metabolic flux, since the ratios are not just regulated by the enzyme’s activity itself, but also affected by factors like substrate availability and product clearance, etc, but are used as potential indicators. To reveal the complex metabolic pathways of OCM, interventional studies using isotope tracers that pass through these pathways should be conducted to examine enzyme activity and fluxes through metabolic pathways.

## 5. Conclusions

In this study, we identified the dynamics of OCM during pregnancy, including the intricate interplay of the folate cycle, choline metabolism pathway, methionine cycle, and transsulfuration pathway. The decrease in maternal 5-MTHF status with increasing gestational age may lead to increased Hcy remethylation by BHMT and decreased serum betaine concentrations. Longitudinal evaluation would be necessary because maternal serum SAM concentrations remained consistent during pregnancy, whereas SAH increased with advancing gestational age in months. The states of 5-MTHF and betaine may be associated with transsulfuration flux, highlighting the tHcy/tCys ratio as a potentially more sensitive indicator of 5-MTHF and betaine states than tHcy concentration alone. Maternal OCM status could reflect fetal OCM status. Our findings suggest that maternal OCM may change drastically during pregnancy and may affect the fetus, emphasizing the importance of comprehensive and longitudinal evaluation of OCM during pregnancy.

## Figures and Tables

**Table 1:** Serum concentration of one-carbon metabolism-related metabolites at different blood sampling periods.

		Maternal Blood						Cord Blood (*n* = 121)		Cord Blood/Maternal Blood at Birth Ratio	
		Early Pregnancy (*n* = 146)		Late Pregnancy (*n* = 131)		At Birth (*n* = 116)					
Analytes	Unit	Median	(25th, 75th)	Median	(25th, 75th)	Median	(25th, 75th)	Median	(25th, 75th)	Median	(25th, 75th)
5-MTHF ^†^	nmol/L	32.2 ^a^	(20.3, 52.8)	17.0 ^b^	(11.6, 31.7)	14.1 ^c^	(9.8, 23.2)	44.7 ***	(36.5, 64.2)	3.23	(2.18, 4.42)
FA ^†^	nmol/L	0.620 ^a^	(0.095, 1.221)	0.620	(0.127, 1.205)	0.433 ^b^	(0 ^†^, 1.052)	0.530	(0 ^†^, 1.043)	0.90	(0.39, 1.47)
Choline	µmol/L	7.39 ^a^	(6.35, 9.00)	7.98 ^b^	(6.83, 9.80)	11.30 ^c^	(9.45, 12.84)	28.25 ***	(25.05, 32.35)	2.56	(2.18, 3.06)
Betaine	µmol/L	21.1 ^a^	(17.3, 25.3)	13.8 ^b^	(11.8, 17.1)	13.5 ^b^	(11.6, 16.0)	26.9 ***	(24.2, 31.2)	1.95	(1.67, 2.30)
DMG	µmol/L	1.77 ^a^	(1.24, 2.43)	1.70 ^a^	(1.13, 2.44)	2.22 ^b^	(1.64, 3.32)	3.17 ***	(2.57, 4.12)	1.36	(1.16, 1.70)
Betaine/DMG	µmol/L	11.71 ^a^	(9.11, 16.20)	8.37 ^b^	(6.25, 11.93)	6.04 ^c^	(4.40, 8.40)	8.64 ***	(6.63, 11.57)	-	
Methionine	µmol/L	18.7	(16.4, 23.8)	19.2	(17, 22.8)	20.5	(17.9, 23.5)	29.8 ***	(27.6, 33.3)	1.49	(1.32, 1.67)
SAM	nmol/L	59.2	(49.5, 67.6)	58.6	(50.7, 67.2)	60.2	(51.0, 69.3)	113.5 ***	(99.9, 129.8)	1.91	(1.65, 2.23)
SAH	nmol/L	11.2 ^a^	(9.4, 13.5)	12.4 ^b^	(10.0, 14.4)	23.8 ^c^	(18.8, 30.5)	45.3 ***	(38.2, 55.4)	1.89	(1.58, 2.31)
SAM/SAH	µmol/L	5.34 ^a^	(4.11 6.18)	4.74 ^b^	(3.88, 5.84)	2.70 ^c^	(1.88, 3.37)	2.56	(1.95, 3.14)	-	
tHcy ^†^	µmol/L	5.38 ^a^	(4.58, 6.36)	5.61 ^b^	(4.74, 6.96)	7.16 ^c^	(5.88, 9.16)	6.02 ***	(5.01, 7.75)	0.85	(0.76, 0.95)
Homocysteic acid	µmol/L	0 ^‡^	(0 ^‡^,0 ^‡^)	0 ^‡^	(0 ^‡^,0 ^‡^)	0 ^‡^	(0 ^‡^,0 ^‡^)	0 ^‡^	(0 ^‡^,0 ^‡^)	-	
Cystathionine	nmol/L	103 ^a^	(76, 133)	213 ^b^	(165, 287)	214 ^b^	(171, 291)	327 ***	(245, 402)	1.37	(1.18, 1.68)
tCys	µmol/L	240 ^a^	(219, 258)	213 ^b^	(199, 229)	241 ^a^	(218, 264)	213 ***	(197, 231)	0.90	(0.82, 0.97)
tHcy/tCys	µmol/L	0.0230 ^a^	(0.0194, 0.0259)	0.0268 ^b^	(0.0231, 0.0322)	0.0302 ^c^	(0.0246, 0.0355)	0.0295 ***	(0.0236, 0.0347)	-	
Taurine	µmol/L	66.7 ^a^	(54.6, 95.6)	60.0 ^b^	(48.1, 77.5)	75.1 ^a^	(51.9, 105.3)	187.0 ***	(146.5, 230.8)	2.55	(1.80, 3.76)
Serine	µmol/L	99 ^a^	(88, 110)	104 ^b^	(91, 115)	114 ^c^	(98, 129)	156 ***	(143, 169)	1.35	(1.23, 1.54)
Glycine	µmol/L	153 ^a^	(139, 174)	151 ^a^	(131, 172)	172 ^b^	(146, 207)	260 ***	(235, 283)	1.51	(1.30, 1.72)
Riboflavin	nmol/L	9.92 ^a^	(2.32, 18.18)	7.20 ^b^	(1.99, 17.65)	7.98 ^b^	(2.69, 14.59)	55.65 ***	(34.13, 79.28)	5.63	(3.41, 9.56)
Pyridoxamine	nmol/L	0.218	(0.170, 0.265)	0.220	(0.175, 0.269)	0.233	(0.195, 0.305)	0.325 ***	(0.267, 0.433)	1.37	(1.02, 1.85)
Pyridoxine	nmol/L	0.135	(0.092, 0.205)	0.127	(0.072, 0.188)	0.124	(0.072, 0.169)	0.211 ***	(0.139, 0.355)	1.74	(1.09, 3.59)

Values are presented as the median with 25th and 75th percentiles. Different letters denote statistically significant differences between early and late pregnancy and at birth (Wilcoxon signed-rank test with Bonferroni correction, *p* < 0.0167; *n* = 113). *** *p* < 0.0001 (Wilcoxon signed-rank test) when comparing maternal blood at birth versus cord blood (*n* = 114). The cord blood/maternal blood at birth ratio is calculated as maternal blood at birth divided by cord blood (*n* = 114). ^†^ Previously reported analytes [19]. ^‡^ below the detection limit. The *p*-values for the Wilcoxon signed-rank test are summarized in Supplementary Table S1. Abbreviations: 5-MTHF, 5-methyltetrahydrofolate; FA, folic acid; DMG, dimethylglycine; SAM, S-adenosylmethionine; SAH, S-adenosylhomocysteine; tHcy, total homocysteine; tCys, total cysteine.

**Table 2:** Correlation of serum OCM-related metabolite concentrations between maternal and cord blood samples at birth.

Analytes	ρ	*p*-Value
5-MTHF ^†^	0.688	<0.0001
FA ^†^	0.372	<0.0001
Choline	0.397	<0.0001
Betaine	0.366	<0.0001
DMG	0.811	<0.0001
Methionine	0.466	<0.0001
SAM	0.390	<0.0001
SAH	0.386	<0.0001
tHcy ^†^	0.828	<0.0001
Cystathionine	0.593	<0.0001
tCys	0.570	<0.0001
Taurine	0.169	0.0730
Serine	0.345	0.0002
Glycine	0.579	<0.0001
Riboflavin	0.677	<0.0001
Pyridoxamine	0.261	0.0051
Pyridoxine	0.411	<0.0001

Spearman correlation coefficient ρ, and *p*-value. ^†^ Previously reported analytes [19]. Abbreviations: 5-MTHF, 5-methyltetrahydrofolate; FA, folic acid; DMG, dimethylglycine; SAM, S-adenosylmethionine; SAH, S-adenosylhomocysteine; tHcy, total homocysteine; tCys, total cysteine.

**Table 3:** Correlation of serum 5-methyltetrahydrofolate, betaine concentration, betaine/dimethylglycine ratio with S-adenosylmethionine, S-adenosylhomocysteine, total homocysteine concentration, and total homocysteine/total cysteine ratio during blood sampling periods.

	Maternal Blood												Cord Blood			
	Early Pregnancy				Late Pregnancy				At Birth							
	SAM	SAH	tHcy ^†^	tHcy/tCys	SAM	SAH	tHcy ^†^	tHcy/tCys	SAM	SAH	tHcy ^†^	tHcy/tCys	SAM	SAH	tHcy ^†^	tHcy/tCys
5-MTHF ^†^	0.207 *	0.075	−0.356 *	−0.505 *	0.284 *	0.099	−0.518 *	−0.626 *	0.217 *	0.104	−0.544 *	−0.670 *	0.257 *	0.000	−0.394 *	−0.472
Betaine	0.258 *	0.116	−0.241 *	−0.254 *	0.429 *	0.140	−0.355 *	−0.413 *	0.362 *	0.196 *	−0.224 *	−0.339 *	0.333 *	0.099	−0.100	−0.193
Betaine/DMG	−0.094	−0.232 *	−0.168 *	−0.218 *	0.128	−0.163	−0.372 *	−0.340 *	0.074	−0.011	−0.545 *	−0.509 *	0.180 *	−0.027	−0.486 *	−0.458

Values in the correlation matrix represent Spearman correlation coefficients, and asterisks indicate the statistical significance of the correlation coefficient *p* < 0.05. Sample sizes for maternal blood in early pregnancy (*n* = 146), late pregnancy (*n* = 131), at delivery (*n* = 116), and cord blood (*n* = 121) are provided with no missing values. ^†^ Previously reported analytes [19]. Abbreviations: 5-MTHF, 5-methyltetrahydrofolate; FA, folic acid; DMG, dimethylglycine; SAM, S-adenosylmethionine; SAH, S-adenosylhomocysteine; tHcy, total homocysteine; tCys, total cysteine.

## Data Availability

The raw data supporting the conclusions of this article will be made available by the authors upon request.

## Supplementary Materials

The following supporting information can be downloaded at: https://www.mdpi.com/article/10.3390/nu16111765/s1, Supplementary Table S1. *p*-values for the Wilcoxon signed-rank test in Table 1; Supplementary Table S2. Correlation matrices between serum one-carbon metabolism-related substance concentrations in each blood sampling period; Supplementary Figure S1. Correlation of *S*-adenosylmethionine and *S*-adenosylhomocysteine between maternal blood in early, late pregnancy, and at delivery.
