# Supplementary material for: Longitudinal Analysis of One-Carbon Metabolism-Related Metabolites in Maternal and Cord Blood of Japanese Pregnant Women
Source: Nutrients. 2024 Jun 4;16(11):1765. doi: 10.3390/nu16111765 (PMC11174998; doi:10.3390/nu16111765)
Supplement: Supplementary file 1 [file nutrients-16-01765-s001.zip › nutrients-2999129-supplementary.pdf]

Supplementary Table S1. p- values for the Wilcoxon signed-rank test in Table 1.

	Multiple comparisons of maternal blood samples by period of collection			Maternal blood at birth vs. cord blood
	Early vs. late pregnancy	Early pregnancy vs. at birth	Late pregnancy vs. at birth	
5-MTHF†	<0.0001	<0.0001	<0.0001	<0.0001
FA†	0.7582	0.0114	0.0433	0.1871
Choline	0.0002	<0.0001	<0.0001	<0.0001
Betaine	<0.0001	<0.0001	0.2208	<0.0001
DMG	0.7958	<0.0001	<0.0001	<0.0001
Betaine/DMG	<0.0001	<0.0001	<0.0001	<0.0001
Methionine	0.2401	0.0413	0.0496	<0.0001
SAM	0.5160	0.5216	0.8332	<0.0001
SAH	0.0025	<0.0001	<0.0001	<0.0001
SAM/SAH	0.0011	<0.0001	<0.0001	0.9608
tHcy†	0.0144	<0.0001	<0.0001	<0.0001
Homocysteic acid	-	-	-	-
Cystathionine	<0.0001	<0.0001	0.8488	<0.0001
tCys	<0.0001	0.7477	<0.0001	<0.0001
tHcy/tCys	<0.0001	<0.0001	<0.0001	<0.0001
Taurine	0.0013	0.7124	0.0036	<0.0001
Serine	0.0028	<0.0001	<0.0001	<0.0001
Glycine	0.0231	<0.0001	<0.0001	<0.0001
Riboflavin	0.0012	0.0027	0.4250	<0.0001
Pyridoxamine	0.8611	0.0417	0.0908	<0.0001
Pyridoxine	0.8868	0.6073	0.5078	<0.0001

† Previously reported analytes [19]. -, homocysteic acid was not statistically analyzed because all samples were below the limit of quantitation.

Supplementary Table S2. Correlation matrices between serum one-carbon metabolism-related substance concentrations in each blood sampling period [maternal blood in early pregnancy (A), in late pregnancy (B), at delivery (C) and cord blood (D)].

(A) Maternal Blood in Early Pregnancy

	Folate Cycle		Choline Metabolic Pathways				Methionine Cycle					Transsulfuration Pathway				Amino Acids		Cofactor		
	5-MTHF†	FA†	Choline	Betaine	DMG	Betaine/DMG	Methionine	SAM	SAH	SAM/SAH	tHcy†	Cystathionine	tCys	tHcy/tCys	Taurine	Serine	Glycine	Riboflavin	Pyridoxamine	Pyridoxine
5-MTHF†	-	0.348	0.098	0.090	-0.105	0.163	0.055	0.207	0.075	0.055	-0.356	-0.199	0.200	-0.505	0.210	-0.123	0.023	0.270	0.200	0.094
FA†	0.348	-	-0.087	0.045	-0.147	0.180	-0.139	-0.027	-0.010	0.026	-0.122	-0.197	0.094	-0.184	-0.053	-0.160	-0.119	0.138	0.081	0.081
Choline	0.098	-0.087	-	0.573	0.439	-0.133	0.250	0.379	0.481	-0.229	-0.061	0.371	0.051	-0.099	0.286	0.124	0.107	0.194	-0.045	0.128
Betaine	0.090	0.045	0.573	-	0.499	0.006	0.155	0.258	0.116	0.032	-0.241	0.186	0.036	-0.254	-0.027	0.138	0.104	0.104	-0.034	0.046
DMG	-0.105	-0.147	0.439	0.499	-	-0.826	0.357	0.228	0.282	-0.139	0.066	0.299	0.005	0.083	0.100	0.263	0.113	0.057	0.011	0.210
Betaine/DMG	0.163	0.180	-0.133	0.006	-0.826	-	-0.279	-0.094	-0.232	0.164	-0.168	-0.162	0.037	-0.218	-0.097	-0.168	-0.041	0.043	-0.007	-0.209
Methionine	0.055	-0.139	0.250	0.155	0.357	-0.279	-	0.603	0.176	0.210	0.016	0.629	0.130	-0.087	0.144	0.600	0.348	0.180	0.033	0.010
SAM	0.207	-0.027	0.379	0.258	0.228	-0.094	0.603	-	0.178	0.437	-0.113	0.529	0.205	-0.238	0.058	0.133	0.163	0.146	-0.009	-0.091
SAH	0.075	-0.010	0.481	0.116	0.282	-0.232	0.176	0.178	-	-0.762	0.258	0.074	0.159	0.158	0.371	0.175	0.175	0.236	-0.022	0.023
SAM/SAH	0.055	0.026	-0.229	0.032	-0.139	0.164	0.210	0.437	-0.762	-	-0.311	0.238	0.001	-0.311	-0.342	-0.096	-0.056	-0.103	0.002	-0.056
tHcy†	-0.356	-0.122	-0.061	-0.241	0.066	-0.168	0.016	-0.113	0.258	-0.311	-	0.079	0.373	0.847	0.034	0.225	0.180	-0.166	-0.096	0.014
Cystathionine	-0.199	-0.197	0.371	0.186	0.299	-0.162	0.629	0.529	0.074	0.238	0.079	-	-0.091	0.109	-0.039	0.297	0.243	-0.030	-0.156	-0.007
tCys	0.200	0.094	0.051	0.036	0.005	0.037	0.130	0.205	0.159	0.001	0.373	-0.091	-	-0.123	0.216	0.120	0.006	0.139	0.103	0.008
tHcy/tCys	-0.505	-0.184	-0.099	-0.254	0.083	-0.218	-0.087	-0.238	0.158	-0.311	0.847	0.109	-0.123	-	-0.092	0.151	0.167	-0.284	-0.155	0.015
Taurine	0.210	-0.053	0.286	-0.027	0.100	-0.097	0.144	0.058	0.371	-0.342	0.034	-0.039	0.216	-0.092	-	0.259	0.097	0.233	0.120	0.241
Serine	-0.123	-0.160	0.124	0.138	0.263	-0.168	0.600	0.133	0.175	-0.096	0.225	0.297	0.120	0.151	0.259	-	0.505	0.102	-0.026	0.068
Glycine	0.023	-0.119	0.107	0.104	0.113	-0.041	0.348	0.163	0.175	-0.056	0.180	0.243	0.006	0.167	0.097	0.505	-	-0.036	-0.177	0.079
Riboflavin	0.270	0.138	0.194	0.104	0.057	0.043	0.180	0.146	0.236	-0.103	-0.166	-0.030	0.139	-0.284	0.233	0.102	-0.036	-	-0.111	0.101
Pyridoxamine	0.200	0.081	-0.045	-0.034	0.011	-0.007	0.033	-0.009	-0.022	0.002	-0.096	-0.156	0.103	-0.155	0.120	-0.026	-0.177	-0.111	-	0.028
Pyridoxine	0.094	0.081	0.128	0.046	0.210	-0.209	0.010	-0.091	0.023	-0.056	0.014	-0.007	0.008	0.015	0.241	0.068	0.079	0.101	0.028	-

(B) Maternal Blood in Late Pregnancy

	Folate Cycle		Choline Metabolic Pathways				Methionine Cycle					Transsulfuration Pathway				Amino Acids		Cofactor		
	**5-MTHF†**	**FA†**	**Choline**	**Betaine**	**DMG**	**Betaine/DMG**	**Methionine**	**SAM**	**SAH**	**SAM/SAH**	**tHcy†**	**Cystathionine**	**tCys**	**tHcy/tCys**	**Taurine**	**Serine**	**Glycine**	**Riboflavin**	**Pyridoxamine**	**Pyridoxine**
5-MTHF†	-	0.224	0.255	0.393	-0.123	0.293	0.253	0.284	0.099	0.084	-0.518	-0.088	0.171	-0.626	0.107	-0.108	0.220	0.295	0.118	0.005
FA†	0.224	-	0.005	0.129	0.021	0.040	0.046	0.089	0.116	-0.074	-0.148	-0.033	-0.020	-0.117	0.198	0.088	0.039	0.089	-0.003	0.201
Choline	0.255	0.005	-	0.640	0.312	-0.046	0.420	0.417	0.391	-0.081	-0.130	0.191	0.196	-0.207	0.311	0.262	0.163	0.009	0.077	0.009
Betaine	0.393	0.129	0.640	-	0.336	0.085	0.427	0.429	0.140	0.133	-0.355	0.159	0.088	-0.413	0.109	0.212	0.144	0.149	0.066	-0.004
DMG	-0.123	0.021	0.312	0.336	-	-0.889	0.214	0.091	0.176	-0.124	0.200	0.096	0.175	0.145	0.041	0.334	-0.083	0.024	0.040	0.019
Betaine/DMG	0.293	0.040	-0.046	0.085	-0.889	-	-0.002	0.128	-0.163	0.238	-0.372	-0.024	-0.155	-0.340	-0.004	-0.239	0.156	0.070	-0.018	0.005
Methionine	0.253	0.046	0.420	0.427	0.214	-0.002	-	0.448	0.195	0.078	-0.175	0.364	0.048	-0.219	0.071	0.415	0.336	0.091	0.059	-0.048
SAM	0.284	0.089	0.417	0.429	0.091	0.128	0.448	-	0.149	0.492	-0.176	0.214	0.208	-0.292	-0.008	0.011	0.116	0.038	0.179	-0.020
SAH	0.099	0.116	0.391	0.140	0.176	-0.163	0.195	0.149	-	-0.748	0.190	0.126	0.150	0.131	0.414	0.224	0.163	0.078	0.052	0.107
SAM/SAH	0.084	-0.074	-0.081	0.133	-0.124	0.238	0.078	0.492	-0.748	-	-0.258	0.061	0.025	-0.289	-0.339	-0.185	-0.084	-0.065	0.082	-0.143
tHcy†	-0.518	-0.148	-0.130	-0.355	0.200	-0.372	-0.175	-0.176	0.190	-0.258	-	0.019	0.358	0.892	0.115	0.330	0.122	-0.323	-0.008	0.075
Cystathionine	-0.088	-0.033	0.191	0.159	0.096	-0.024	0.364	0.214	0.126	0.061	0.019	-	-0.003	0.015	0.033	0.187	0.047	0.008	-0.051	-0.216
tCys	0.171	-0.020	0.196	0.088	0.175	-0.155	0.048	0.208	0.150	0.025	0.358	-0.003	-	-0.051	0.172	0.215	0.137	0.010	0.077	0.074
tHcy/tCys	-0.626	-0.117	-0.207	-0.413	0.145	-0.340	-0.219	-0.292	0.131	-0.289	0.892	0.015	-0.051	-	0.085	0.278	0.059	-0.348	-0.037	0.026
Taurine	0.107	0.198	0.311	0.109	0.041	-0.004	0.071	-0.008	0.414	-0.339	0.115	0.033	0.172	0.085	-	0.375	0.175	-0.075	0.019	0.102
Serine	-0.108	0.088	0.262	0.212	0.334	-0.239	0.415	0.011	0.224	-0.185	0.330	0.187	0.215	0.278	0.375	-	0.404	-0.115	0.094	-0.025
Glycine	0.220	0.039	0.163	0.144	-0.083	0.156	0.336	0.116	0.163	-0.084	0.122	0.047	0.137	0.059	0.175	0.404	-	-0.065	-0.100	0.095
Riboflavin	0.295	0.089	0.009	0.149	0.024	0.070	0.091	0.038	0.078	-0.065	-0.323	0.008	0.010	-0.348	-0.075	-0.115	-0.065	-	-0.206	0.140
Pyridoxamine	0.118	-0.003	0.077	0.066	0.040	-0.018	0.059	0.179	0.052	0.082	-0.008	-0.051	0.077	-0.037	0.019	0.094	-0.100	-0.206	-	-0.057
Pyridoxine	0.005	0.201	0.009	-0.004	0.019	0.005	-0.048	-0.020	0.107	-0.143	0.075	-0.216	0.074	0.026	0.102	-0.025	0.095	0.140	-0.057	-

(C) Maternal Blood at Birth

	Folate Cycle		Choline Metabolic Pathways				Methionine Cycle					Transsulfuration Pathway				Amino Acids		Cofactor		
	5-MTHF†	FA†	Choline	Betaine	DMG	Betaine/DMG	Methionine	SAM	SAH	SAM/SAH	tHcy†	Cystathionine	tCys	tHcy/tCys	Taurine	Serine	Glycine	Riboflavin	Pyridoxamine	Pyridoxine
5-MTHF†	-	0.090	0.171	0.338	-0.329	0.516	0.100	0.217	0.104	0.008	-0.544	-0.267	0.177	-0.670	0.134	-0.145	0.100	0.296	0.108	0.086
FA†	0.090	-	-0.086	0.053	-0.112	0.154	-0.163	0.083	-0.224	0.167	-0.149	-0.237	-0.099	-0.080	0.097	-0.218	-0.254	0.000	-0.117	-0.184
Choline	0.171	-0.086	-	0.490	0.203	0.005	0.386	0.007	0.574	-0.390	-0.058	0.170	0.208	-0.140	0.331	0.417	0.481	0.011	0.233	0.059
Betaine	0.338	0.053	0.490	-	0.275	0.180	0.399	0.362	0.196	0.071	-0.224	0.247	0.223	-0.339	0.183	0.241	0.184	0.122	0.184	-0.039
DMG	-0.329	-0.112	0.203	0.275	-	-0.864	0.177	0.081	0.131	-0.013	0.427	0.263	0.254	0.338	-0.048	0.287	0.058	-0.119	0.091	-0.145
Betaine/DMG	0.516	0.154	0.005	0.180	-0.864	-	0.017	0.074	-0.011	0.006	-0.545	-0.203	-0.154	-0.509	0.110	-0.203	0.009	0.187	0.025	0.152
Methionine	0.100	-0.163	0.386	0.399	0.177	0.017	-	0.192	0.293	-0.070	-0.003	0.265	0.120	-0.046	0.126	0.516	0.460	0.088	0.188	0.003
SAM	0.217	0.083	0.007	0.362	0.081	0.074	0.192	-	-0.186	0.640	-0.019	0.181	0.137	-0.058	0.113	-0.157	-0.192	0.072	0.022	0.094
SAH	0.104	-0.224	0.574	0.196	0.131	-0.011	0.293	-0.186	-	-0.823	0.026	0.171	0.107	-0.034	0.192	0.334	0.512	-0.026	0.235	0.020
SAM/SAH	0.008	0.167	-0.390	0.071	-0.013	0.006	-0.070	0.640	-0.823	-	0.001	0.037	0.024	0.013	-0.081	-0.265	-0.465	0.038	-0.157	0.002
tHcy†	-0.544	-0.149	-0.058	-0.224	0.427	-0.545	-0.003	-0.019	0.026	0.001	-	0.186	0.423	0.886	0.012	0.249	0.056	-0.245	0.005	-0.042
Cystathionine	-0.267	-0.237	0.170	0.247	0.263	-0.203	0.265	0.181	0.171	0.037	0.186	-	0.107	0.126	-0.027	0.284	0.143	-0.203	-0.131	-0.005
tCys	0.177	-0.099	0.208	0.223	0.254	-0.154	0.120	0.137	0.107	0.024	0.423	0.107	-	0.024	0.225	0.234	0.150	0.082	0.133	0.104
tHcy/tCys	-0.670	-0.080	-0.140	-0.339	0.338	-0.509	-0.046	-0.058	-0.034	0.013	0.886	0.126	0.024	-	-0.087	0.167	-0.010	-0.332	-0.065	-0.080
Taurine	0.134	0.097	0.331	0.183	-0.048	0.110	0.126	0.113	0.192	-0.081	0.012	-0.027	0.225	-0.087	-	0.388	0.196	0.116	-0.044	0.073
Serine	-0.145	-0.218	0.417	0.241	0.287	-0.203	0.516	-0.157	0.334	-0.265	0.249	0.284	0.234	0.167	0.388	-	0.627	-0.053	0.118	-0.099
Glycine	0.100	-0.254	0.481	0.184	0.058	0.009	0.460	-0.192	0.512	-0.465	0.056	0.143	0.150	-0.010	0.196	0.627	-	-0.046	0.143	0.046
Riboflavin	0.296	0.000	0.011	0.122	-0.119	0.187	0.088	0.072	-0.026	0.038	-0.245	-0.203	0.082	-0.332	0.116	-0.053	-0.046	-	0.096	0.183
Pyridoxamine	0.108	-0.117	0.233	0.184	0.091	0.025	0.188	0.022	0.235	-0.157	0.005	-0.131	0.133	-0.065	-0.044	0.118	0.143	0.096	-	0.057
Pyridoxine	0.086	-0.184	0.059	-0.039	-0.145	0.152	0.003	0.094	0.020	0.002	-0.042	-0.005	0.104	-0.080	0.073	-0.099	0.046	0.183	0.057	-

(D) Cord Blood

	Folate Cycle		Choline Metabolic Pathways				Methionine Cycle					Transsulfuration Pathway				Amino Acids		Cofactor		
	**5-MTHF†**	**FA†**	**Choline**	**Betaine**	**DMG**	**Betaine/DMG**	**Methionine**	**SAM**	**SAH**	**SAM/SAH**	**tHcy†**	**Cystathionine**	**tCys**	**tHcy/tCys**	**Taurine**	**Serine**	**Glycine**	**Riboflavin**	**Pyridoxamine**	**Pyridoxine**
5-MTHF†	-	0.208	0.171	0.253	-0.226	0.353	0.140	0.257	0.000	0.099	-0.394	-0.097	0.207	-0.472	-0.007	0.014	0.210	0.296	0.127	-0.098
FA†	0.208	-	0.075	0.061	-0.015	0.034	-0.206	-0.075	-0.077	-0.010	-0.049	-0.183	0.025	-0.047	-0.058	-0.118	-0.127	-0.010	-0.107	-0.046
Choline	0.171	0.075	-	0.514	0.230	0.077	0.324	-0.002	0.245	-0.197	0.058	-0.042	0.067	0.015	0.318	0.161	0.306	0.165	0.084	-0.088
Betaine	0.253	0.061	0.514	-	0.311	0.230	0.272	0.333	0.099	0.072	-0.100	0.024	0.223	-0.193	0.101	0.217	0.179	0.145	0.083	0.061
DMG	-0.226	-0.015	0.230	0.311	-	-0.812	0.067	0.010	0.063	-0.064	0.406	0.230	0.181	0.329	0.015	0.256	0.027	-0.172	0.029	-0.021
Betaine/DMG	0.353	0.034	0.077	0.230	-0.812	-	0.102	0.180	-0.027	0.135	-0.486	-0.269	-0.055	-0.458	0.046	-0.086	0.105	0.299	-0.006	0.023
Methionine	0.140	-0.206	0.324	0.272	0.067	0.102	-	0.319	0.138	0.046	0.014	0.267	0.307	-0.066	0.186	0.432	0.506	0.254	0.251	0.179
SAM	0.257	-0.075	-0.002	0.333	0.010	0.180	0.319	-	-0.187	0.651	0.041	0.380	0.301	-0.072	0.051	0.202	0.153	0.112	0.097	0.002
SAH	0.000	-0.077	0.245	0.099	0.063	-0.027	0.138	-0.187	-	-0.820	0.140	0.085	0.091	0.093	0.301	0.276	0.269	0.005	0.142	0.171
SAM/SAH	0.099	-0.010	-0.197	0.072	-0.064	0.135	0.046	0.651	-0.820	-	-0.079	0.119	0.051	-0.097	-0.208	-0.158	-0.164	0.023	-0.058	-0.128
tHcy†	-0.394	-0.049	0.058	-0.100	0.406	-0.486	0.014	0.041	0.140	-0.079	-	0.291	0.209	0.895	0.082	0.224	0.035	-0.343	-0.118	-0.160
Cystathionine	-0.097	-0.183	-0.042	0.024	0.230	-0.269	0.267	0.380	0.085	0.119	0.291	-	0.113	0.263	0.120	0.243	0.124	-0.069	0.108	0.141
tCys	0.207	0.025	0.067	0.223	0.181	-0.055	0.307	0.301	0.091	0.051	0.209	0.113	-	-0.174	0.076	0.368	0.353	0.187	0.114	-0.132
tHcy/tCys	-0.472	-0.047	0.015	-0.193	0.329	-0.458	-0.066	-0.072	0.093	-0.097	0.895	0.263	-0.174	-	0.039	0.104	-0.086	-0.441	-0.168	-0.106
Taurine	-0.007	-0.058	0.318	0.101	0.015	0.046	0.186	0.051	0.301	-0.208	0.082	0.120	0.076	0.039	-	0.279	0.251	0.180	0.241	0.236
Serine	0.014	-0.118	0.161	0.217	0.256	-0.086	0.432	0.202	0.276	-0.158	0.224	0.243	0.368	0.104	0.279	-	0.589	0.162	0.205	0.159
Glycine	0.210	-0.127	0.306	0.179	0.027	0.105	0.506	0.153	0.269	-0.164	0.035	0.124	0.353	-0.086	0.251	0.589	-	0.262	0.188	0.124
Riboflavin	0.296	-0.010	0.165	0.145	-0.172	0.299	0.254	0.112	0.005	0.023	-0.343	-0.069	0.187	-0.441	0.180	0.162	0.262	-	0.032	0.126
Pyridoxamine	0.127	-0.107	0.084	0.083	0.029	-0.006	0.251	0.097	0.142	-0.058	-0.118	0.108	0.114	-0.168	0.241	0.205	0.188	0.032	-	0.461
Pyridoxine	-0.098	-0.046	-0.088	0.061	-0.021	0.023	0.179	0.002	0.171	-0.128	-0.160	0.141	-0.132	-0.106	0.236	0.159	0.124	0.126	0.461	-

Values in the correlation matrix are Spearman correlation coefficient, $p < 0.05$, with positive correlations shown in blue and negative correlations shown in red. Serum homocysteic acid, pyridoxamine, and pyridoxine concentrations were below the limit of quantitation in all samples, so results are not shown.Sample sizes

were maternal blood in early pregnancy (n = 146), in late pregnancy (n = 131), at delivery (n = 116) and cord blood (n = 121) with no missing values. † Previously reported analytes [19].Abbreviations, 5-MTHF; 5-methyltetrahydrofolate; FA; folic acid; DMG; dimethylglycine; SAM; S-adenosylmethionine; SAH; S-adenosylhomocysteine; tHcy; total homocysteine; tCys; total cysteine.

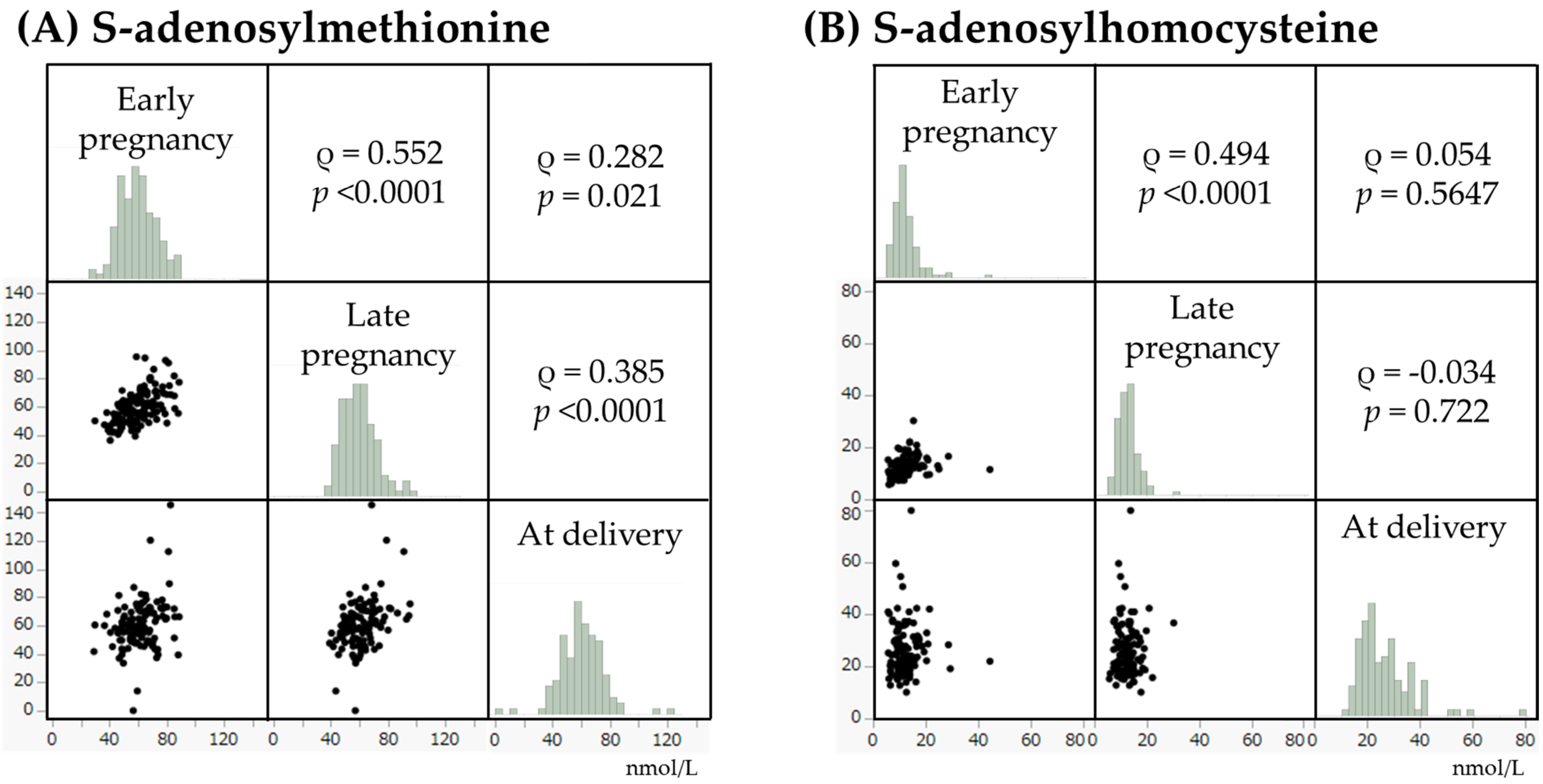

Supplementary Figure S1. Correlation of *S*-adenosylmethionine and *S*-adenosylhomocysteine between maternal blood in early, late pregnancy, and at delivery. Spearman correlation coefficient ϱ, and p-value. Upper right shows Spearman correlation coefficient and p-value. Lower left is a scatter plot of the correlation

coefficients shown in the upper right. The sample size was in early and in late pregnancy (n = 131), in early pregnancy and at delivery (n = 116) and in late pregnancy and at delivery (n = 113).
